# The Most Potent Natural Pharmaceuticals, Cosmetics, and Food Ingredients Isolated from Plants with Deep Eutectic Solvents

**DOI:** 10.1021/acs.jafc.3c01656

**Published:** 2023-07-11

**Authors:** Agata Wawoczny, Danuta Gillner

**Affiliations:** † Department of Organic Chemistry, Bioorganic Chemistry and Biotechnology, Faculty of Chemistry, Silesian University of Technology, 44-100 Gliwice, Poland; ‡ Biotechnology Centre, Silesian University of Technology, 44-100 Gliwice, Poland

**Keywords:** bioactive compounds from plants, bioactive ingredients in cosmetics, natural pharmaceuticals, natural food additives, deep eutectic solvents, green solvents

## Abstract

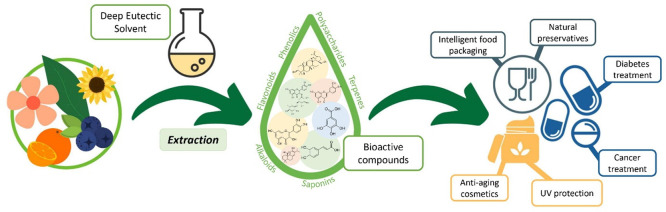

There is growing interest in reducing the number of synthetic products or additives and replacing them with natural ones. The pharmaceutical, cosmetic, and food industries are especially focused on natural and bioactive chemicals isolated from plants or microorganisms. The main challenge here is to develop efficient and ecological methods for their isolation. According to the strategies and rules of sustainable development and green chemistry, green solvents and environmentally friendly technologies must be used. The application of deep eutectic solvents as efficient and biodegradable solvents seems to be a promising alternative to traditional methods. They are classified as being green and ecological but, most importantly, very efficient extraction media compared to organic solvents. The aim of this review is to present the recent findings on green extraction, as well as the biological activities and the possible applications of natural plant ingredients, namely, phenolics, flavonoids, terpenes, saponins, and some others. This paper thoroughly reviews modern, ecological, and efficient extraction methods with the use of deep eutectic solvents (DESs). The newest findings, as well as the factors influencing the efficiency of extraction, such as water content, and hydrogen bond donor and acceptor types, as well as the extraction systems, are also discussed. New solutions to the major problem of separating DESs from the extract and for solvent recycling are also presented.

## 1 Introduction

**Figure 1:**
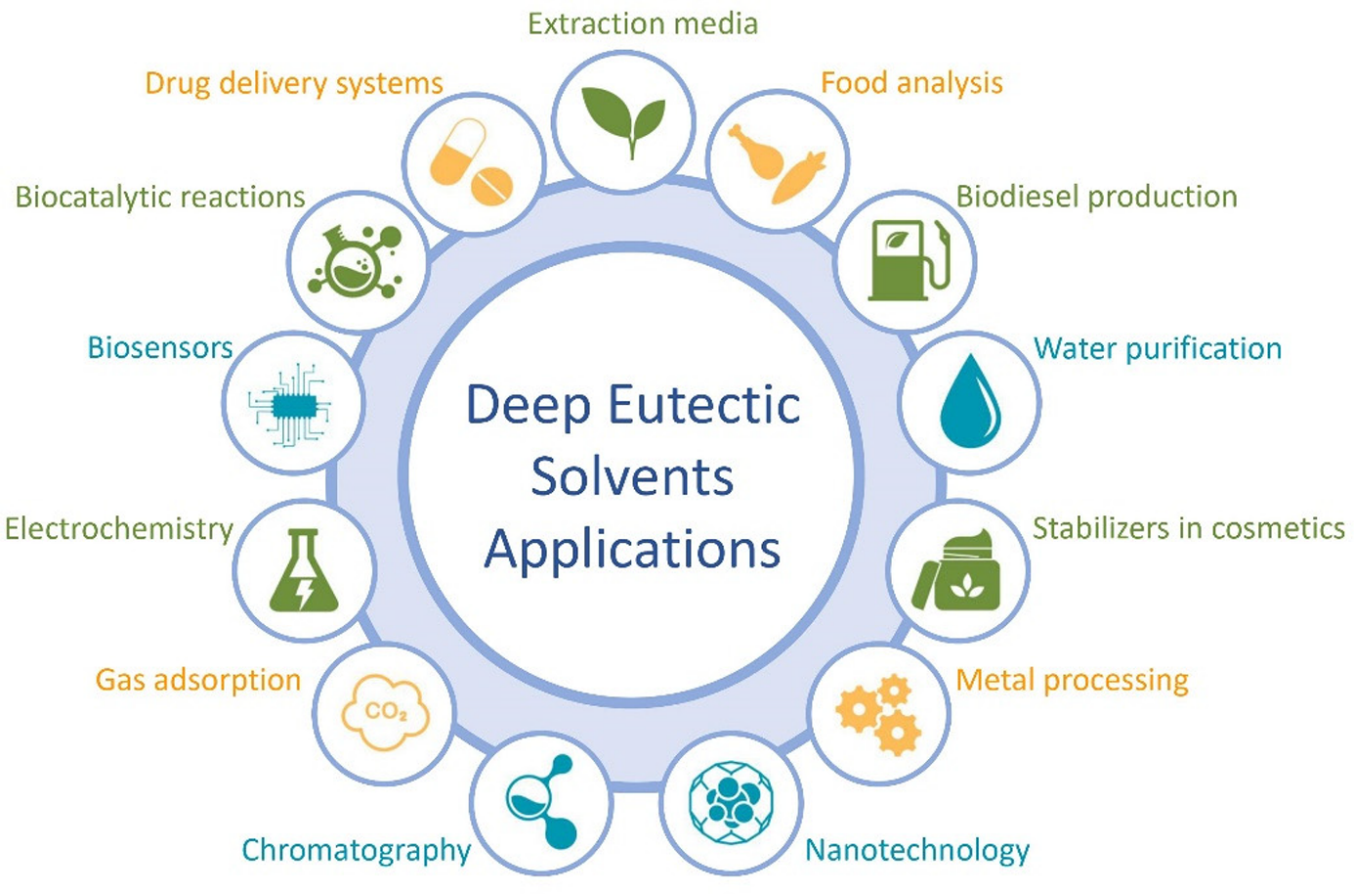
Wide range of DES applications.^4−16^

Nowadays, reducing global pollution and introducing sustainable development in all areas of life should be everyone’s priority. A sustainable development strategy to save the planet and the environment consists of many goals, and one of the most important is responsible production and consumption. A growing amount of interest and demand for natural components with promising properties such as antiaging, anti-inflammatory, anticancer, and antimicrobial has been observed in the pharmaceutical, cosmetic, and food industries. The most interesting groups of valuable, plant-originated compounds include phenolics, flavonoids, terpenoids, and saponins. The major concern involves efficient and ecological isolation of these compounds from natural sources. The technologies applied should meet the requirements of green chemistry, which means that the use of many organic solvents is restricted. There are still many attempts to find green, efficient, and inexpensive methods for the isolation of natural plant ingredients, both hydrophilic and hydrophobic. The new trend, widely explored in many fields of industry, is to apply deep eutectic solvents (DESs), composed of naturally occurring ingredients. These have been proven to be efficient, biodegradable, and environmentally friendly media, useful in many branches of industry (Figure 1).^1−16^

**Figure 2:**
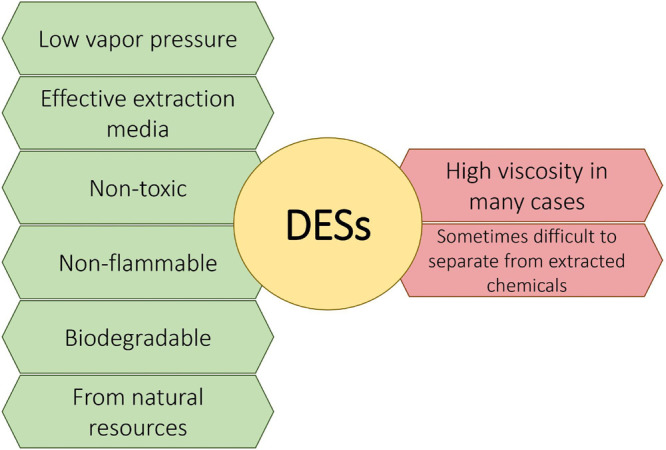
Advantages (green) and disadvantages (red) of deep eutectic solvents applied as extraction media.

DESs were first introduced in 2003 by Abbott et al., and since then, huge development and interest in these solvents have been observed.^17^ They have been considered as potential replacements for ionic liquids (ILs), after aspects of the possible toxicity of some ILs to living organisms have emerged. Initially, the research focused mostly on hydrophilic DESs, and the first information on hydrophobic ones appeared in 2015. The structures of the most popular DESs are based on noncovalent interactions. Depending on the character of the hydrogen bond acceptor (HBA) and the hydrogen bond donor (HBD), interactions such as hydrogen bonding, van der Waals interactions, or π–π stacking occur. Recently, novel eutectic systems, namely, eutectic molecular liquids (EMLs), were defined, and several other noncovalent interactions were identified, including not only hydrogen bonding and π–π stacking but also σ-hole (halogen, pnicogen, and tetrel bonds), π-hole, κ-hole, and μ-hole bonding interactions.^18,19^ There is a huge interest in composing DESs with new and interesting properties as well as in explaining the structure–activity relationship and the interactions occurring between different HBAs and HBDs.^20−22^ The most valuable feature of DESs seems to be the possibility of obtaining almost unlimited numbers of tailor-made solvents by selecting appropriate HBDs and HBAs. Additionally, many DESs (also named natural deep eutectic solvents, NADESs) are bioderived, green, biocompatible, biodegradable, and nontoxic chemicals, unlike classic solvents (including solvents belonging to volatile organic compounds, VOCs) or some ILs. However, there are a few negative aspects for DES applications. First is the high viscosity of many DESs. Also, due to the high hygroscopicity of some DESs (especially those with choline chloride), they can absorb moisture from the air, which may cause problems with handling and storage. Additionally, even though the majority of DESs are considered thermostable, some can decompose at high temperatures and some can be corrosive.^23^ The major challenge in using DESs as extraction media is to efficiently separate them from the extract and to recycle them for reuse. The main advantages and disadvantages of DESs as extraction media are presented in Figure 2. It is clear that the advantages far outweigh the disadvantages, so DESs are much better and more preferable substitutes for organic solvents.

Many papers discussing the extractions of plant ingredients with DESs have been published in recent times. These focus on several important aspects of efficient extraction, including the types and properties of the solvents, the time of extraction, and the water content and temperature.^24,25^ Different extraction techniques, e.g., microwave- and ultrasound-assisted extraction and microextraction, have been reviewed.^26−28^ The analytical methods applied to the characteristics of the extracted substances, as well as the problems with the viscosity and recovery of the extracted compounds, are also widely discussed.^29,30^ Special attention is paid to the extraction of phenolic compounds, flavonoids, proteins, biopolymers, and carbohydrates from waste biomass or algae.^24,31,32^ Among all of the types of DESs used in modern extraction, the potential for hydrophobic DESs was underlined in many recent papers, and thoroughly reviewed by Sportiello et al.^33^ These DESs can be characterized by the presence of compounds with long alkyl chain or cycloalkyl groups. They are composed of, e.g., lauric acid, decanoic acid, thymol, menthol, tetraheptylammonium chloride, or tetraoctylammonium chloride as HBAs and long chain carboxylic acids, levulinic acid, lidocaine, or camphor as HBDs. Efficient extraction of food contaminants, chelating properties, and also extraction of bioactive compounds such as artemisinin, carotenoids, or tocols are widely reported.^33^ The importance of the extraction of natural substances with DESs is also confirmed by the number of patent applications filed in recent years. According to the Espacenet database, in only 2022–2023, out of 91 documents claiming extractions using DESs, ∼67 patent applications were filed for the extraction of natural compounds (https://worldwide.espacenet.com/; query: deep eutectic solvent AND extraction; selected by earliest publication date; accessed 2023-10-05). The newest inventions, apart from the classic DESs, focus on extractions with NADESs, hydrophobic DESs, binary solvents (e.g., composed of ILs and DESs), or deep eutectic solvent microemulsion extraction systems, as well as the use of ultrasound- or microwave-assisted processes.

This review provides a compilation of the newest information on the significance of the most important groups of bioactive compounds from plants, namely, phenolics, flavonoids, terpenes, saponins, polysaccharides, and some others. It focuses on the novel findings of their biological activities, the new potential applications of the extracted chemicals, and ecologically sound and efficient extraction methods with the use of DESs. The advantage of applying DESs, as well as the influence of their structures on the efficiency of extraction, is thoroughly discussed. Additionally, technological aspects, including the influence of additives, the extraction system, and conditions, are discussed. The newest solutions of the crucial problem concerning solute recovery after extraction and DES recycling are also presented. Further perspectives, taking into account the strengths and weaknesses of extraction using DESs, are also discussed.

## 2 The Most Interesting Bioactive Compounds Extracted with DESs from Plant Material

The most interesting, widely investigated plant ingredients belong to one of the following groups: phenolics, flavonoids, terpenoids, saponins, polysaccharides, or alkaloids. Each group contains many bioactive compounds with interesting properties, which makes them promising chemicals for the pharmaceutical, cosmetic, and food industries. In this section, the properties and potencies of the compounds most frequently mentioned in the recent literature are presented.

### 2.1 Phenolic Compounds

Phenolic compounds can be successfully extracted from plants occurring in all habitats. It is also worth mentioning that the majority of them are already known for their interesting properties that are useful in medicine, foods, or cosmetics. They are edible, e.g., found in tomatoes, olives, grapes, common purslane, and common knotgrass; or they are part of the group of herbs, e.g., mint, lavender, and *Plantago asiatica*.^34,35^ The antimicrobial activities and perspectives of phenolic applications as food preservatives or biocides are widely discussed.^36^ Phenolics such as chlorogenic acid, gallic acid, caffeic acid, and rosmarinic acid are secondary metabolites in plants.^37^ Because of their antioxidant and anti-inflammatory properties, they can be used as active ingredients in the cosmetic, food, and pharmaceutical industries.^38−40^Table 1 presents the newest findings in the bioactivities and possible future applications of the chosen phenolic compounds extracted from plants.^41−86^ Based on the information presented in Table 1, it is clear that current research focuses mostly on the medical application of phenolics in anticancer preparations. It is worth mentioning that phenolics have shown activities in the treatment of other civilization and neurodegenerative diseases.

**Table 1:**
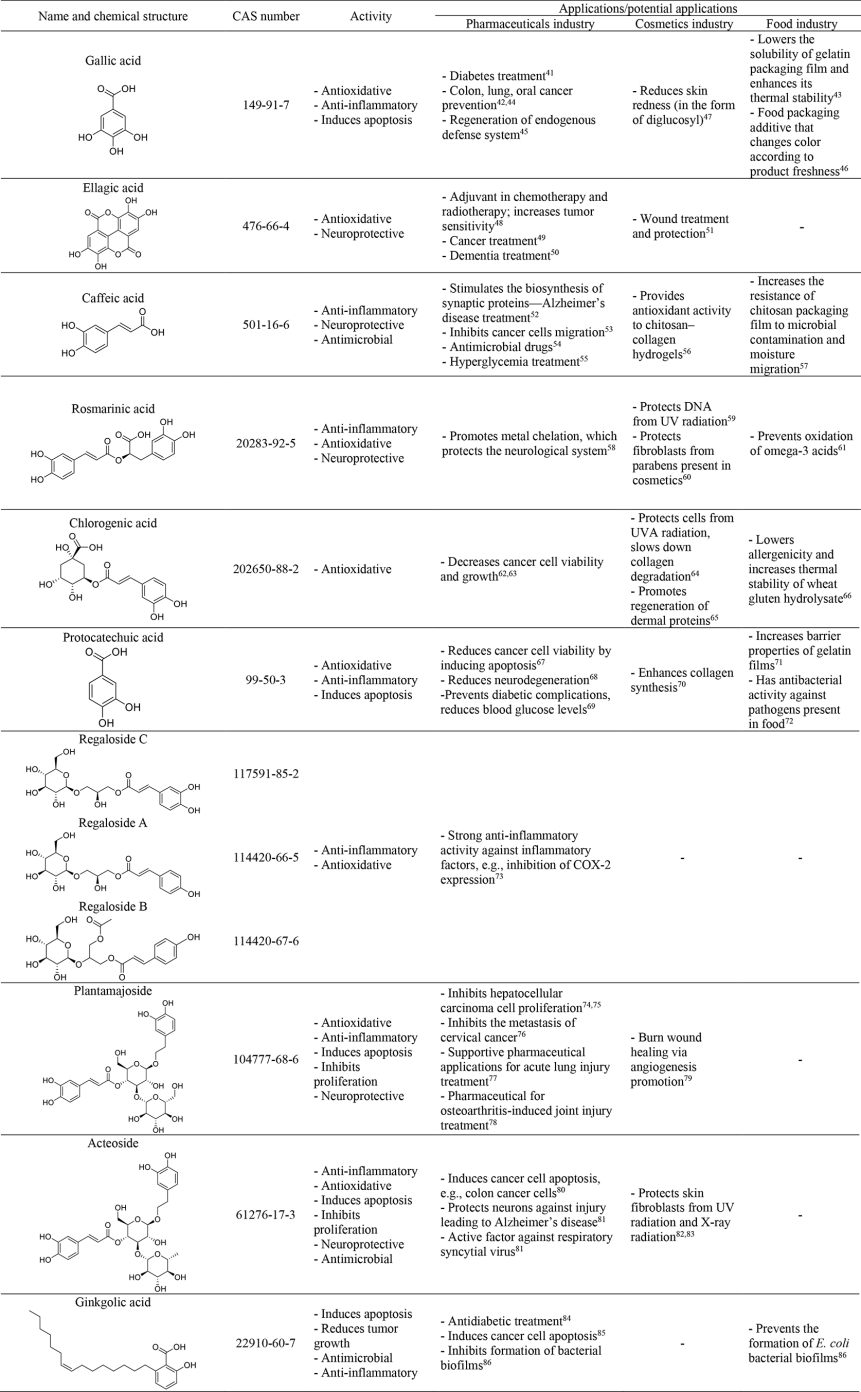
Chemical Structures, Properties, and Application of Phenolic Compounds Present in Plants

The extraction of phenolic compounds has been widely discussed in the literature. Scientists are attempting to replace classic methods with more ecologically sound methods. An overview of the newest findings in the extraction of these valuable compounds from different types of plant materials using DESs is presented in Table 2. Several factors (the molar ratio of the HBD and HBA in DES, the method of extraction, the temperature, water addition, and the time of extraction) influencing the efficiency of the process are included.^87−108^

**Table 2:** Extraction of Phenolic Compounds Using Deep Eutectic Solvents^a^

biomass (part)	DES	molar ratio	method of extraction	temp [°C]	water content [%]	time [min]	extracted compd	yield (calcd on 1 g of dry biomass)	ref
*Vitis rotundifolia* (fruit skins)	ChCl:lactic acid	1:2	UAE	60	20	30	ellagic acid	21300 μg/g	(87)
	ChCl:oxalic acid	1:1	UAE	60	30	30	ellagic acid	16000 μg/g	
	ChCl:lactic acid	1:2	UAE	60	20	30	gallic acid	10300 μg/g	
	ChCl:oxalic acid	1:1	UAE	60	30	30	gallic acid	10400 μg/g	
*Olea europaea* L. (leaves)	ChCl:acetic acid	1:1	SAE	50	20	180	total phenolic content	470 μg GA/g^b^	(88)
	ChCl:malic acid	1:1	SAE	50	20	180	total phenolic content	260 μg GA/g^b^	
	ChCl:malonic acid	1:1	SAE	50	20	180	total phenolic content	250 μg GA/g^b^	
	ChCl:citric acid	2:1	SAE	50	20	180	total phenolic content	240 μg GA/g^b^	
*Solanum lycopersicum* (fruit peels)	ChCl:1,2-propanediol	1:2	UAE	65	10	60	caffeic acid	2 μg/g	(89)
	ChCl:1,2-propanediol	1:2	UAE	65	10	60	chlorogenic acid	37 μg/g	
	ChCl:1,2-propanediol	1:2	UAE	65	10	60	gallic acid	10 μg/g	
	ChCl:lactic acid	1:2	UAE	65	10	60	caffeic acid	5 μg/g	
	ChCl:lactic acid	1:2	UAE	65	10	60	chlorogenic acid	52 μg/g	
	ChCl:lactic acid	1:2	UAE	65	10	60	gallic acid	14 μg/g	
*Apocynum ventem* L. (leaves)	ChCl:levulinic acid	1:2	SAE	50	45	30	gallic acid	1009 μg/g	(90)
	ChCl:levulinic acid	1:2	SAE	50	45	30	protocatechuic acid	418 μg/g	
	ChCl:levulinic acid	1:2	MAE	–	45	0.5	gallic acid	924 μg/g	
	ChCl:levulinic acid	1:2	MAE	–	45	0.5	protocatechuic acid	575 μg/g	
Brewer’s spent grain	ChCl:glycerol	1:2	MAE	–	25	20	total phenolic content	3780 μg GA/g^b^	(91)
	ChCl:ethylene glycol	1:2	MAE	–	25	20	total phenolic content	2150 μg GA/g^b^	
*Leptospermum scoparium* (leaves)	ChCl:ethylene glycol	1:2	SAE	RT	0	60	total phenolic content	56870 μg GA/g^b^	(92)
	ChCl:1,3-propanediol	1:3	SAE	RT	0	60	total phenolic content	50670 μg GA/g^b^	
	ChCl:lactic acid	1:2	SAE	RT	0	60	total phenolic content	52520 μg GA/g^b^	
	ChCl:acetic acid	1:2	SAE	RT	0	60	total phenolic content	32490 μg GA/g^b^	
*Lavandula angustifolia* (flowers)	ChCl:glycerol	1:2	UAE	40	30	30	total phenolic content	44650 μg GA/g^b^	(93)
	ChCl:glycerol	1:3	UAE	40	30	30	total phenolic content	41460 μg GA/g^b^	
	ChCl:glycerol	1:4	UAE	40	30	30	total phenolic content	40750 μg GA/g^b^	
*Lilium lancifolium* Thunb. (bulbs)	ChCl:ethylene glycol	1:2	SAE	50	20	40	regaloside C	310 μg/g	(94)
	ChCl:ethylene glycol	1:2	SAE	50	20	40	regaloside E	290 μg/g	
	ChCl:ethylene glycol	1:2	SAE	50	20	40	regaloside B	3040 μg/g	
*Plantago asiatica* L. (aerial part)	ChCl:lactic acid:ethylene glycol	1:4:2	UAE	60	50	35	plantamajoside	3830 μg/g	(95)
	ChCl:lactic acid:ethylene glycol	1:4:2	UAE	60	50	35	acteoside	4230 μg/g	
*Sanghuangporus baumii* (fruiting body)	ChCl:malic acid	1:2	UAE	50	40	30	total phenolic content	6370 μg GA/g^b^	(96)
*Polygonum aviculare* (leaves)	ChCl:levulinic acid	1:2	UAE	70	38	60	gallic acid	39800 μg/g	(97)
	ChCl:levulinic acid	1:2	UAE	70	38	60	5-caffeoylquinic acid	11830 μg/g	
	ChCl:levulinic acid	1:2	UAE	70	38	60	chlorogenic acid	7160 μg/g	
*Eucommia ulmoides* (leaves)	ChCl:1,4-butanediol:ascorbic acid	1:1:0.1	MAE	–	20	20	chlorogenic acid	3659 μg/g	(98)
*Achillea millefolium* L. (aerial parts)	ChCl:lactic acid	1:2	UAE	50	25	30	chlorogenic acid	2970 μg/g	(99)
	ChCl:urea	2:1						2900 μg/g	
*Mentha piperita* (leaves)	ChCl:glycerol	1:1	SAE	60	30	30	chlorogenic acid	160 μg/g	(100)
	ChCl:glycerol	1:1	SAE	60	30	30	rosmarinic acid	3233 μg/g	
	ChCl:citric acid	1:1	SAE	60	30	30	chlorogenic acid	206 μg/g	
	ChCl:citric acid	1:1	SAE	60	30	30	rosmarinic acid	12005 μg/g	
*Rhus coriaria* L. (fruits)	ChCl:lactic acid	1:2	UAE	25	20	30	total phenolic content	124960 μg GA/g^b^	(101)
	ChCl:acetic acid	1:2	UAE	25	20	30	total phenolic content	119570 μg GA/g^b^	
*Ginkgo biloba* (leaves)	ChCl:ascorbic acid	1:1	UAE	70	30	30	ginkgolic acid	10434 μg/g	(102)
	ChCl:fructose	1:1	UAE	70	30	30	ginkgolic acid	8155 μg/g	
*Malus domestica* (fruit pomace)	ChCl:glycerol	1:2	UAE	30	30	30	total phenolic content	5600 μg GA/g^b^	(103)
	ChCl:citric acid	1:1	UAE	30	30	30	total phenolic content	4600 μg GA/g^b^	
*Mentha piperita* (leaves)	ChCl:glycerol	1:2	SAE	70	20	180	rosmarinic acid	5090 μg/g	(104)
*Curcuma longa* L. (rhizomes)	ChCl:citric acid	1:1	MAE	30	30	6	curcuminoids	89870 μg/g	(105)
*Phoenix dactylifera* L. (seeds)	ChCl:lactic acid	1:2	UAE	40	30	15	total phenolic content	145540 μg GA/g^b^	(106)
*Cosmos sulphureus* (seeds)	ChCl:lactic acid	1:2	UAE	47.5	32.6	40	total phenolic content	35300 μg GA/g^b^	(107)
*Curcuma longa* L. (rhizomes)	ChCl:malonic acid	1:1	UAE	90	50	60	curcuminoids	165510 μg/g	(108)
	ChCl:propionic acid	1:2	UAE	90	50	60	curcuminoids	120920 μg/g	
	ChCl:oxalic acid	1:1	UAE	90	50	60	curcuminoids	112210 μg/g	
	ChCl:ethylene glycol	1:2	UAE	90	50	60	curcuminoids	50870 μg/g	

a RT, room temperature; GA, gallic acid; UAE, ultrasound-assisted extraction; MAE, microwave-assisted extraction; SAE, stirring-assisted extraction.

b Mass of all phenolic compounds, calculated as mass of the gallic acid.

The yields of extracted chemicals depend on a few factors. The major factor is the type of plant and the contents of the active compounds. However, some trends in the influences of other factors can also be observed. When the type of HBD in the DES is considered, the application of an acidic HBD, rather than polyalcohols and sugars, is more favorable. For instance, in an extraction from *Solanum lycopersicum*, higher yields of gallic acid, chlorogenic acid, and caffeic acid were obtained while using DES ChCl:lactic acid, compared to ChCl:1,2-propanediol (for gallic acid, the difference was 4 μg/g; for chlorogenic acid, 15 μg/g; and for caffeic acid, 3 μg/g).^89^ The same phenomenon was observed in the isolation of rosmarinic acid from *Mentha piperita*. The DES ChCl:citric acid was much more efficient than ChCl:glycerol (the yield was 8800 μg/g higher).^99^ Similar results were observed by Alrugaibah et al.^87^ The extraction of ellagic acid from *Vitis rotundifolia* was much more efficient with DES ChCl:lactic acid (21.3 mg/g of dry biomass) and ChCl:oxalic acid (16.0 mg/g), compared to ChCl:1,2-propanediol (8.4 mg/g).

The other important issue in the extraction of phenolics with DESs is the chosen technique. As can be seen in Table 2, ultrasound- or microwave-assisted processes allow for a reduced time of extraction and a high yield of the desired chemical to be obtained. For example, in the microwave extraction of protocatechuic acid from *Apocynum ventem* L. with the DES ChCl:levulinic acid, a yield of 575 μg/g was obtained in 30 s, while traditional stirring extraction required 30 min to obtain 418 μg/g.^90^

### 2.2 Flavonoids

Flavonoids can also be considered as phenolic compounds, but because of their distinctive chemical structures, based on 2-phenyl-chromone, they are presented separately.^109^ Similar to phenolics, flavonoids can be found in plants from all over the world, especially in edible ones. The richest sources are fruits from *Prunus* (e.g., cherry and plum) and *Vaccinium* (e.g., cranberry and blueberry) species. There have also been many attempts to utilize byproducts or wastes from the forestry and food industries. An interesting example is the utilization of bark from *Larix decidua* (European larch), which is a byproduct of timber production. Studies revealed that it can be a promising and cheap source of flavonoids.^110,111^ Flavonoids have great potential as anticancer and anti-inflammatory pharmaceuticals, as well as antiaging agents in cosmetics. Examples of these compounds, extracted from plants with DESs, are presented in Table 3.^46,112−181^

**Table 3:**
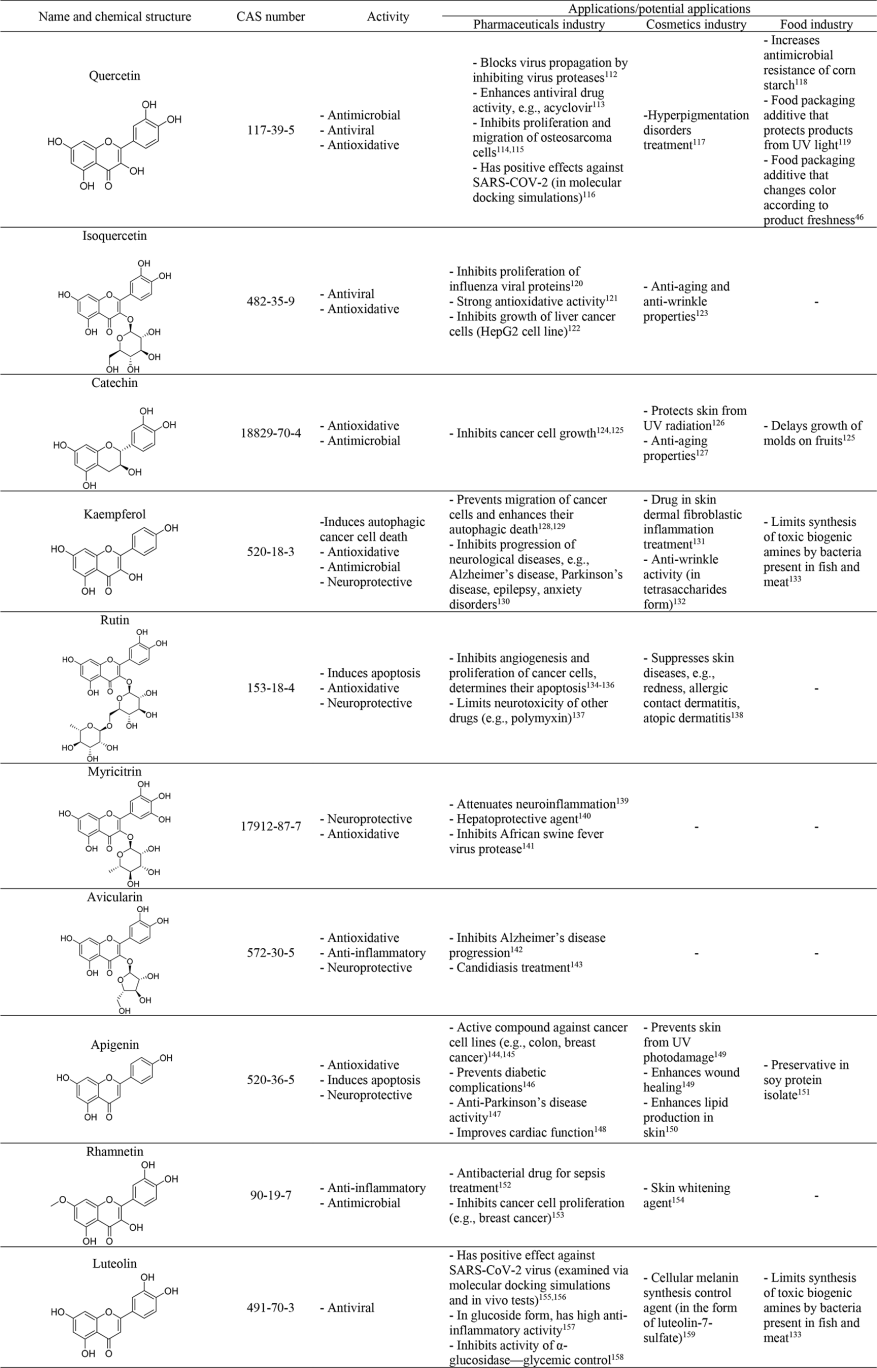

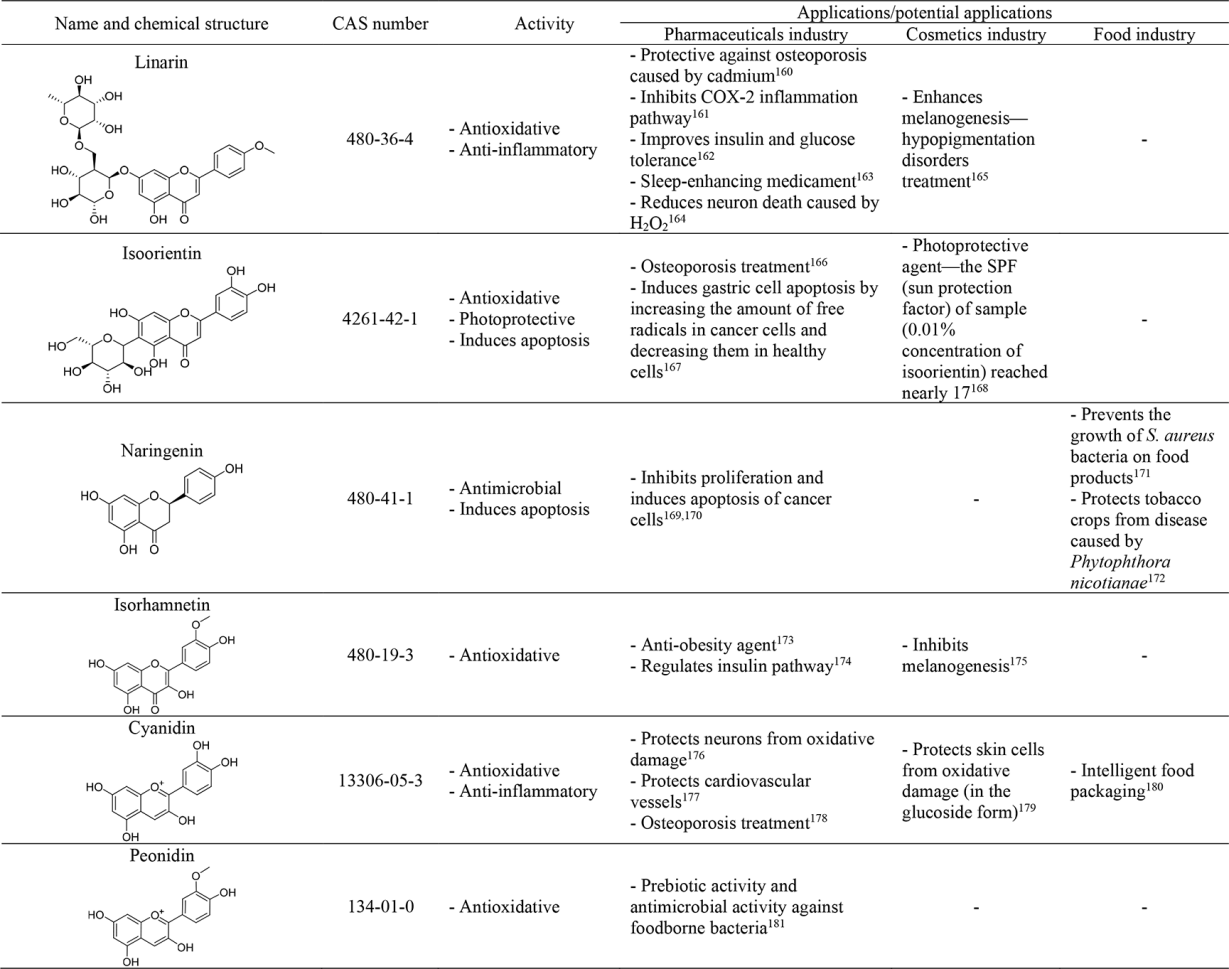
Chemical Structures, Properties, and Application of Flavonoids Present in Plants

Flavonoids are commonly presented as glycosides, such as cyanidin-3-*O*-glucoside and quercetin 3-rutinoside, because of their increased efficiencies as pharmaceuticals. After glycosylation their water solubilities and stabilities are enhanced, which improves their bioavailability.^182^

Natural flavonoids have also been studied as additives/preservatives in the food industry. They have very good antioxidative and antimicrobial properties, which makes them promising natural preservatives. Examples of flavonoid extraction with DESs from plant material are presented in Table 4.^89,90,95,97,98,100,104,183−196^

**Table 4:** Extraction of Flavonoids Using Deep Eutectic Solvents

biomass	DES	molar ratio	method of extraction^a^	temp [°C]	water content [%]	time [min]	extracted compd	yield (calcd on 1 g of dry biomass)	ref
*Polygonatum odoratum* (rhizomes)	ChCl:malic acid	1:1	UAE	50	0	20	total flavonoid content	4760 μg/g	(183)
	ChCl:citric acid	1:1	UAE	50	0	20	total flavonoid content	6830 μg/g	
	ChCl:lactic acid	1:1	UAE	50	0	20	total flavonoid content	7320 μg/g	
*Larix decidua* (bark)	ChCl:urea	1:2	SAE	58	75	94	total flavonoid content	159000 μg/g	(184)
	ChCl:urea	1:2	SAE	58	50	94	total flavonoid content	305000 μg/g	
	ChCl:urea	1:2	SAE	58	25	94	total flavonoid content	275000 μg/g	
	ChCl:1,4-butanediol	1:2	SAE	58	75	94	total flavonoid content	376000 μg/g	
	ChCl:1,4-butanediol	1:2	SAE	58	50	94	total flavonoid content	376000 μg/g	
	ChCl:1,4-butanediol	1:2	SAE	58	25	94	total flavonoid content	383000 μg/g	
*Polygonatum sibiricum* (whole plant)	ChCl:acetic acid	1:4	UAE	50	30	30	total flavonoid content	3000 μg/g	(185)
*Solanum lycopersicum* (fruit peels)	ChCl:1,2-propanediol	1:2	UAE	65	10	60	quercetin	1.02 μg/g	(89)
	ChCl:lactic acid	1:2	UAE	65	10	60	quercetin	1.47 μg/g	
*Apocynum ventem* L. (leaves)	ChCl:levulinic acid	1:2	SAE	50	45	30	catechin	4338 μg/g	(90)
	ChCl:levulinic acid	1:2	SAE	50	45	30	rutin	169 μg/g	
	ChCl:levulinic acid	1:2	SAE	50	45	30	isoquercetin	2031 μg/g	
	ChCl:levulinic acid	1:2	MAE	–	45	0.5	catechin	5025 μg/g	
	ChCl:levulinic acid	1:2	MAE	–	45	0.5	rutin	255 μg/g	
	ChCl:levulinic acid	1:2	MAE	–	45	0.5	isoquercetin	3411 μg/g	
*Plantago asiatica* L. (aerial part)	ChCl:lactic acid:ethylene glycol	1:4:2	UAE	60	50	35	quercetin	560 μg/g	(95)
	ChCl:lactic acid:ethylene glycol	1:4:2	UAE	60	50	35	kaempferol	190 μg/g	
*Syzygium cumini* (fruits)	ChCl:citric acid	1:1	MAE	–	40	2.5	total anthocyanine content	7864 μg/g	(186)
	ChCl:citric acid	1:1	UAE	70	40	150	total anthocyanine content	8525 μg/g	
*Polygonum aviculare* (leaves)	ChCl:levulinic acid	1:2	UAE	70	38	60	myricitrin	10470 μg/g	(97)
	ChCl:levulinic acid	1:2	UAE	70	38	60	3″-*O*-galloylmyricitrin	7920 μg/g	
	ChCl:levulinic acid	1:2	UAE	70	38	60	quercitrin	4260 μg/g	
	ChCl:levulinic acid	1:2	UAE	70	38	60	avicularin	3020 μg/g	
*Chrysanthemum indicum* L. (flowers)	ChCl:ethylene glycol	1:2	UAE	RT	30	32	linarin	14230 μg/g	(187)
*Fagopyrum esculentum* (sprouts)	ChCl:urea	1:2	UAE	40	20	40	isoorientin	5110 μg/g	(188)
	ChCl:triethylene glycol	1:4	UAE	40	20	40	isoorientin	6660 μg/g	
	ChCl:urea	1:2	UAE	40	20	40	rutin	1840 μg/g	
	ChCl:triethylene glycol	1:4	UAE	40	20	40	rutin	2220 μg/g	
pollen typhae (pollen)	ChCl:1,2-propanediol	1:4	UAE	RT	30	35	quercetin	383 μg/g	(189)
	ChCl:1,2-propanediol	1:4	UAE	RT	30	35	naringenin	48 μg/g	
	ChCl:1,2-propanediol	1:4	UAE	RT	30	35	kaempferol	391 μg/g	
	ChCl:1,2-propanediol	1:4	UAE	RT	30	35	isorhamnetin	3149 μg/g	
cranberry pomace	ChCl:lactic acid	1:2	UAE	60	20	30	cyanidin-3-galactoside	180 μg/g	(190)
	ChCl:lactic acid	1:2	UAE	60	20	30	peonidin 3-arabinoside	320 μg/g	
	ChCl:lactic acid	1:2	UAE	60	50	30	cyanidin-3-galactoside	300 μg/g	
	ChCl:lactic acid	1:2	UAE	60	50	30	peonidin 3-arabinoside	600 μg/g	
*Eucommia ulmoides* (leaves)	ChCl:1,4-butanediol:ascorbic acid	1:1:0.2	MAE	–	20	20	rutin	1143 μg/g	(98)
	ChCl:1,4-butanediol:ascorbic acid	1:1:0.2	MAE	–	20	20	isoquercetin	1087 μg/g	
*Jaboticaba* processing byproduct	ChCl:propylene glycol	1:2	pressurized liquid extraction	90	53	12	cyanidin-3-*O*-glucoside	29400 μg/g	(191)
	ChCl:malic acid	1:2	pressurized liquid extraction	90	53	12	cyanidin-3-*O*-glucoside	25780 μg/g	
*Buddleja globosa* (leaves)	ChCl:1,2-propanediol	1:3	SAE	60	20	20	luteolin-7-*O*-glucoside	4387 μg/g	(192)
*Mentha piperita* (leaves)	ChCl:glycerol	1:1	SAE	60	30	30	luteolin-7-*O*-glucoside	5856 μg/g	(100)
	ChCl:glycerol	1:1	SAE	60	30	30	apigenin	361 μg/g	
	ChCl:citric acid	1:1	SAE	60	30	30	luteolin 7-*O*-glucoside	7998 μg/g	
	ChCl:citric acid	1:1	SAE	60	30	30	apigenin	384 μg/g	
*Polygonum maritimum* L. (leaves and stems)	ChCl:sucrose	1:2	UAE	RT	30	30	rhamnetin-3-galactoside	1470 μg/g	(193)
sour cherry pomace	ChCl:malic acid	1:1	SAE	40	20	30	quercetin-3-rutinoside	172 μg/g	(194)
	ChCl:malic acid	1:1	UAE	40	20	30	quercetin-3-rutinoside	119 μg/g	
	ChCl:malic acid	1:1	MAE	–	20	0.25	quercetin-3-rutinoside	138 μg/g	
*Potentilla Fruticose* L. (leaves and stems)	ChCl:citric acid	1:1	UAE	28	30	30	catechin-7-*O*-glucoside	12200 μg/g	(195)
*Mentha piperita* (leaves)	ChCl:glycerol	1:2	SAE	70	20	180	hesperidin	3220 μg/g	(104)
*Dendrobium officinale* (stems)	ChCl:lactic acid	1:4	UAE	50	30	40	total flavonoid content	35240 μg/g	(196)

a UAE, ultrasound-assisted extraction; MAE, microwave-assisted extraction; SAE, stirring-assisted extraction.

As can be seen from Table 4, there is no clear tendency for the influence of DES composition on the yield of flavonoids. For example, in the extraction of quercetin from tomatoes, the yield was similar for DES with acidic and diol HBDs (1.02 μg/g for DES ChCl:1,2-propanediol and 1,47 μg/g for ChCl:lactic acid).^89^ In some cases, better results were obtained in an extraction with DES containing diols compared to other HBDs (e.g., the yield of rutin extracted from *Fagopyrum esculentum* with ChCl:triethylene glycol was higher than that extracted with ChCl:urea; the difference was 380 μg/g).^188^

### 2.3 Terpenes, Terpenoids, and Saponins

Another class of compounds with great importance is terpenes and their derivatives—terpenoids are considered to be one of the most diverse groups of plant metabolites.^168^ They are present in many plants and in some marine organisms. A unique feature of all terpenes is a chemical structure based on isoprene molecules. Many of them have pharmacological activities—anticancer, antimicrobial, and anti-inflammatory.^169,170^ Because of their diversity and interesting properties, they have become important ingredients in food, cosmetics, and pharmaceuticals. Their chemical synthesis is usually unprofitable or unsustainable, so efficient extraction from natural sources seems to be a very important direction in research and industrial development.^197−199^Table 5 presents a few examples of terpenes and terpenoids, with their activities and possible applications.^133,200−225^

**Table 5:**
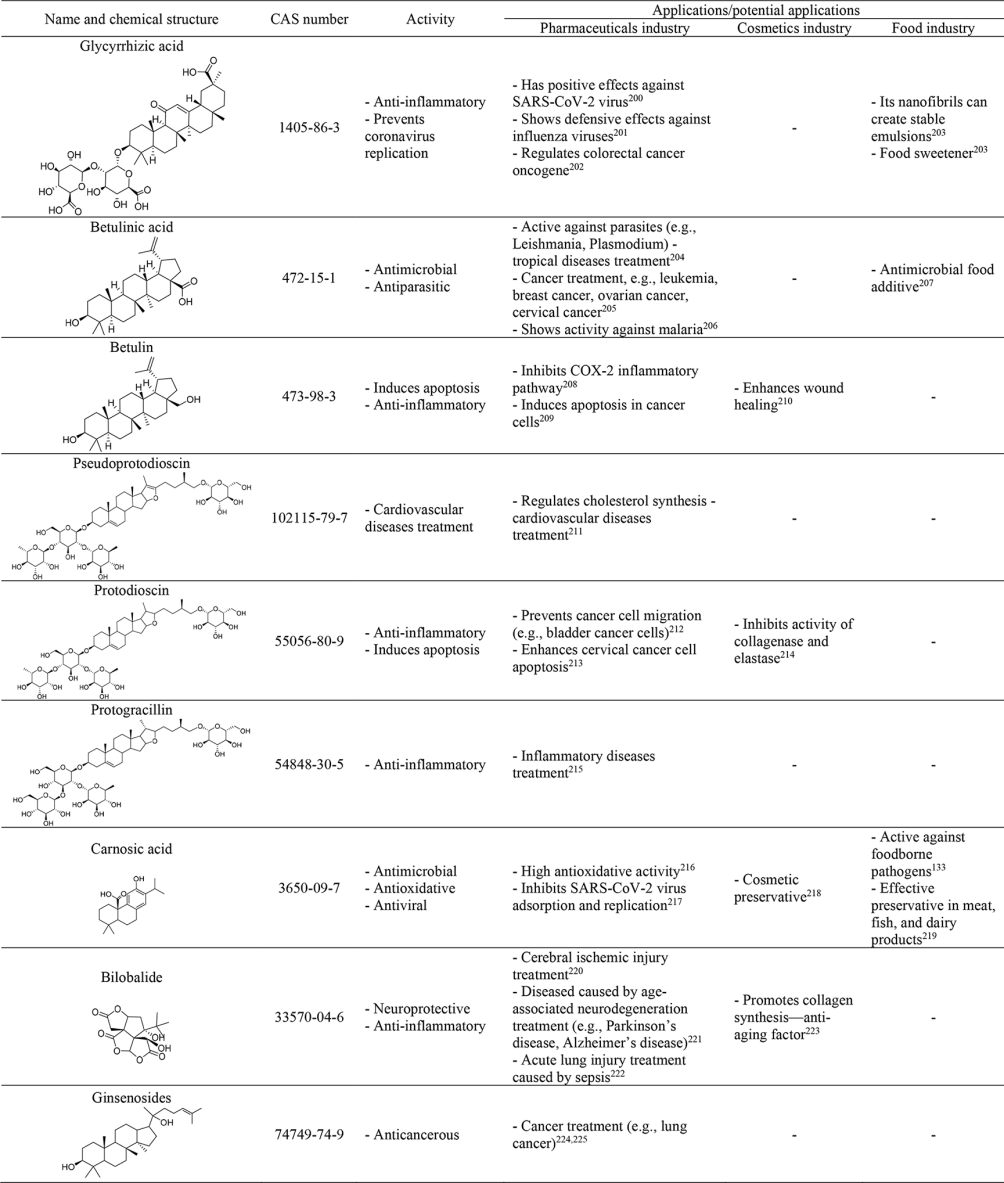
Chemical Structures, Properties, and Application of Terpenes, Terpenoids, and Saponins Present in Plants

The extraction of these groups of compounds from the plant material can be effectively carried out with DESs based on choline chloride as the HBA, summarized in Table 6.^102,226−233^

**Table 6:** Extraction of Terpenes, Terpenoids, and Saponins Using Deep Eutectic Solvents

biomass	DES	molar ratio	method of extraction^a^	temp [°C]	water content [%]	time [min]	extracted compd	yield (calcd on 1 g of dry biomass)	ref
*Glycyrrhiza glabra* (roots)	ChCl:glycerol	1:1	SAE	30	15	60	glycyrrhizic acid	23250 μg/g	(226)
	ChCl:malic acid	1:1	SAE	30	15	60	glycyrrhizic acid	36700 μg/g	
	ChCl:oxalic acid	1:1	SAE	30	15	60	glycyrrhizic acid	39600 μg/g	
	ChCl:lactic acid	1:1	SAE	30	15	60	glycyrrhizic acid	42820 μg/g	
	ChCl:succinic acid	1:1	SAE	30	15	60	glycyrrhizic acid	43650 μg/g	
	ChCl:citric acid	1:1	SAE	30	15	60	glycyrrhizic acid	30670 μg/g	
*Glycyrrhiza glabra* (roots)	ChCl:lactic acid	1:1	SAE	40	30	30	glycyrrhizic acid	53720 μg/g	(227)
*Betula pendula* (bark)	ChCl:lactic acid	1:1	SAE	60	5.4	150	betulinic acid	141 μg/g	(228)
	ChCl:lactic acid	1:1	SAE	80	5.4	150	betulinic acid	263 μg/g	
	ChCl:lactic acid	1:1	SAE	60	5.4	75	betulin	1217 μg/g	
	ChCl:lactic acid	1:1	SAE	60	5.4	105	betulin	1015 μg/g	
	ChCl:lactic acid	1:1	SAE	60	5.4	150	betulin	878 μg/g	
	ChCl:lactic acid	1:1	SAE	80	5.4	75	betulin	1332 μg/g	
	ChCl:lactic acid	1:1	SAE	80	5.4	105	betulin	1788 μg/g	
	ChCl:lactic acid	1:1	SAE	80	5.4	150	betulin	1329 μg/g	
*Dioscorea nipponica* (rhizomes)	ChCl:malonic acid	1:1	UAE	RT	30	20	pseudoprotodioscin	9710 μg/g	(229)
	ChCl:malonic acid	1:1	UAE	RT	30	20	protodioscin	29300 μg/g	
	ChCl:malonic acid	1:1	UAE	RT	30	20	protogracillin	15860 μg/g	
	ChCl:malonic acid	1:1	UAE	RT	30	20	pseudoprotogracillin	3660 μg/g	
*Salvia officinalis* L. (leaves)	ChCl:lactic acid	1:2	SAE	50	10	90	carnosic acid	14.43 μg/g	(230)
	ChCl:lactic acid	1:2	SAE	30	10	60	carnosic acid	10.68 μg/g	
	ChCl:lactic acid	1:2	SAE	70	10	60	carnosic acid	10.16 μg/g	
*Ginkgo biloba*(leaves)	ChCl:ascorbic acid	1:1	UAE	70	30	30	bilobalide	7518 μg/g	(102)
	ChCl:fructose	1:1	UAE	70	30	30	bilobalide	6552 μg/g	
*Xanthoceras sorbifolia* Bunge. (husks)	ChCl:glycolic acid	1:2	SAE	60	30	30	total saponin content	60720 μg/g	(231)
*Panax quinquefolius* L. (roots)	ChCl:ethylene glycol	1:2	SAE	60	20	30	ginsenosides	71590 μg/g	(232)
*Trillium govanianum* (rhizomes)	ChCl:lactic acid	1:1	UAE	50	0	60	total saponin content	114400 μg/g	(233)

a UAE, ultrasound-assisted extraction; MAE, microwave-assisted extraction; SAE, stirring-assisted extraction.

Licorice (*Glycyrrhiza glabra*) is one of the most commonly used plants, because of its high content of glycyrrhizic acid. In the isolation of terpenoids and saponins, the impact of HBDs in applied DESs is similar to the case of phenolic compound extraction: higher yields of products are achieved with DESs when acidic HBDs are used. For example, in a process of bilobalide extraction from *Ginkgo biloba*, extraction with the DES ChCl:ascorbic acid yielded 7518 μg/g product while in the presence of ChCl:fructose 6552 μg/g was obtained.^102^ Similar results were reported by Lanjekar and Rathod—the yield of glycyrrhizic acid obtained from licorice was nearly twice as high when succinic acid was the HBD, compared to DES with glycerol.^226^

### 2.4 Other Chemicals Extracted from Plants with DESs

There are many other interesting bioactive molecules that can be isolated from plants, apart from the mentioned phenolics, flavonoids, terpenoids, and saponins. Many of them present biological activities that are required by industries, especially the pharmaceutical industry. For instance, polysaccharides extracted from *Zizyphus jujube* fruits show prebiotic activity, as tested on bacteria.^234^ Similar properties were demonstrated for polysaccharides from *Lithocarpus litseifolius* leaves. In addition, these compounds could be applied in diabetes treatment.^235^ Polysaccharides extracted from lotus leaves were examined in terms of their antioxidant capacities. Through extraction with DESs, the antioxidant properties of compounds were more intense compared to the extracts isolated with water.^236^ Other interesting natural chemicals are alkaloids with potential anti-inflammatory bioactivities. This group of compounds has many possible applications, such as the improvement of immunological defense and the treatment of cardiovascular and liver diseases.^237−239^ Carotenoids are chemicals that are commonly present in edible plants, but due to their hydrophobic nature, the recent literature focuses on applying hydrophobic DESs in their extraction.^240^Table 7 presents a brief review on other chemicals extracted using DESs.^234,236,241−244^

**Table 7:** Extraction of Other Chemicals Using Deep Eutectic Solvents

biomass	DES	molar ratio	method of extraction	temp [°C]	water content [%]	time [min]	extracted compd	yield (calcd on 1 g of dry biomass)	ref
*Ziziphus jujuba* Mill. (fruits)	ChCl:ethylene glycol	1:3.75	SAE	90	53	120	polysaccharides	63600 μg/g	(234)
*Nelumbo nucifera* Gaertn. (leaves)	ChCl:ethylene glycol	1:3	SAE	90	30	180	polysaccharides	778900 μg/g	(236)
	ChCl:ethylene glycol	1:3	MAE	–	0	6.5	polysaccharides	832000 μg/g	
*Nelumbo nucifera* Gaertn. (leaves)	ChCl:propylene glycol	1:4	UAE	RT	30	30	*O*-nornuciferine	690 μg/g	(241)
	ChCl:propylene glycol	1:4	UAE	RT	30	30	nuciferine	3340 μg/g	
*Peumus boldus* Mol. (leaves)	ChCl:levulinic acid	1:1	SAE	60	20	50	boldine	427 μg/g	(242)
*Phellodendri amurensis* (cortex)	ChCl:citric acid	1:2	UAE	25	30	30	berberine	10820 μg/g	(243)
	ChCl:citric acid	1:2	UAE	25	30	30	palmatine	4740 μg/g	
apricot pulp	ChCl:tartaric acid	2:1	UAE	30	20 (MeOH)	10	β-carotene	413 μg/g	(244)

## 3 Effect of Type of DES and Process Conditions for Extraction Efficiency

### 3.1 Comparison of DESs and Organic Solvents in the Extraction of Bioactive Plant Ingredients

Traditional methods of extraction include Soxhlet extraction with solvents such as ethanol, methanol, and acetone^245^ and extraction with the aid of stirring and heating, commonly used together with the assistance of ultrasound or microwaves.^246−249^ The application of organic solvents in the extraction processes comes with some disadvantages, such as high flammability, high vapor pressure, and, in some cases, toxicity.^250,251^ Additionally, some extracted compounds, e.g., anthocyanins, are prone to degradation at high temperatures. Thus, it is even more difficult to efficiently extract them with organic solvents. Because of that, there is a need for a simple and ecological extraction method under mild conditions.^252^ The application of DESs can be an option for solving the above-mentioned problems.^253^ In many cases, they are more efficient as extraction media, compared with traditional organic solvents.^30,254^ It was shown that higher antioxidant activity was retained by plant extracts obtained using DESs as the extraction media when compared to organic solvents.^255^ Apart from a higher extraction efficiency, DESs can be more selective than organic solvents. By changing the HBA and HBD, it is possible to adjust the polarity of a DES to that of the target compound.^256^ Additionally, extraction with a DES requires less solvent.^30^ A comparison of extraction efficiencies using DESs and organic solvents is presented in Table 8.^88,90,100,102,257,258^

**Table 8:** Advantage of Using DESs over Organic Solvents in Extraction of Bioactive Compounds from Plant Material

biomass	extracted compd	organic solvent	yield [μg/g] (calcd on 1 g of dry biomass)	DES	yield [μg/g] (calcd on 1 g of dry biomass)	ref
*Mentha piperita* (leaves)	chlorogenic acid	ethanol	175.94	ChCl:malonic acid	194.01	(100)
*Apocynum ventem* L. (leaves)	protocatechuic acid	methanol	284.00	ChCl:levulinic acid	418.00	(90)
*Ginkgo biloba* L. (leaves)	bilobalide	ethanol	5100.00	ChCl:ascorbic acid	7520.00	(102)
*Lavandula pedunculata* subsp. *Lusitanica* (aerial parts)	caffeic acid	methanol	81.50	ChCl:urea	248.30	(257)
*Olea europaea* L. (leaves)	total phenolic content	ethanol	16030.00	ChCl:acetic acid	34610.00	(88)
*Carthamus tinctorius* L. (flowers)	hydroxysafflor yellow A	methanol	15540.00	ChCl:urea	22810.00	(258)

The biggest challenge in extraction processes with DESs is the recovery of the extracted chemicals from the solvent. The low vapor pressure of the DES is an advantage from an ecological point of view, but this makes it impossible to separate the solvent from the extract via distillation, which is a common method in organic solvent extraction. In the latest reports, macroporous resins or solid-phase extraction were applied during the separation of extracted compounds from DESs.^259^

### 3.2 Impact of HBA and HBD in DES

**Figure 3:**
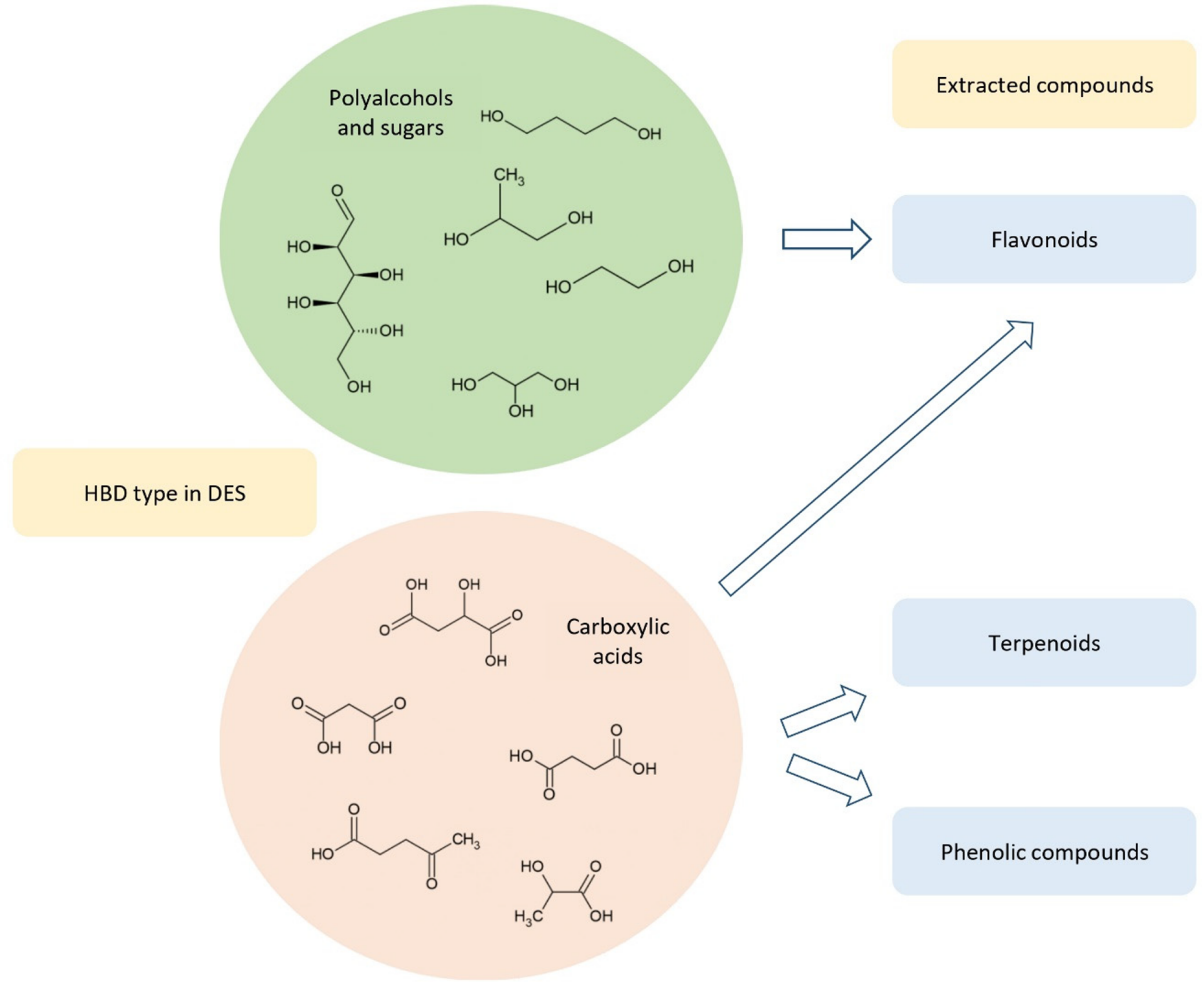
Dependence of HBD in DES and efficiency of extracted metabolites.

As has been mentioned in previous sections, the impact of the HBD on the extraction efficiency depends on the type of the extracted compound. The extraction of flavonoids can be efficiently performed with DESs that possess HBDs such as polyalcohols and organic acids. In the case of phenolic compounds and terpenoids, higher yields of products are obtained in the presence of DESs composed of organic acids as HBDs^102,108,226^ (Figure 3).

This phenomenon is connected with the polarities of DESs and extracted compounds.^260^ The interaction of solute and solvent is indicated by the polarity, which in turn can be studied using solvatochromic data. The E_T_N parameter (normalized polarity) and Kamlet–Taft parameters, namely, α (hydrogen bond donating ability), β (hydrogen bond accepting ability), and π* (dipolarity/polarizability), give information about intermolecular interactions. It has been shown that, based on E_T_N values of DESs having the same HBD (levulinic acid), a hydrophilic HBA makes the solvent more polar. This has been explained by the stability of the hydrogen bond (chloride anion in hydrophilic choline chloride HBA forms a more stable hydrogen bond with HBD than hydrophobic methanol). It can be assumed that, in DESs based on the same HBD, the character of the HBA controls their dipolarity/polarizability as well as the β parameter. Additionally, DESs with carboxylic acids displayed high α values. It was also shown that the addition of water to DESs based on choline chloride and polyalcohols decreases the β parameter and increases π*.^261^ The extraction efficiency can be partially predicted by measuring the αβ value, which represents the strength of hydrogen bonding in the solvent. The efficiency of isolation should increase with higher αβ values.^262^ Many phenolic compounds (e.g., gallic acid and glycyrrhizic acid) are polar, so DESs with higher polarities should be more appropriate for extraction.^263,264^ A study presented by Jurić et al. confirmed that DESs containing organic acids with higher polarities compared to polyalcohols and sugars are better extraction solvents for polar metabolites in the plant material.^265^ In some cases, extraction with polyalcohol-based DESs is more efficient. For instance, the extraction of phenolic compounds from *Leptospermum scoparium* with DES ChCl:ethylene glycol allowed for better yields of phenolics to be obtained (56.87 mg/g of dry biomass, calculated as equivalents of gallic acid) than those with ChCl:lactic acid (52.51 mg/g of dry biomass) or ChCl:acetic acid (32.49 mg/g).^92^ This could be explained by lower viscosities of some DESs with diols as HBDs than with organic acids, which facilitates mass transport during extraction (e.g., the viscosity of ChCl:lactic acid at 25 °C is equal 301.902 mPa·s and that of ChCl:ethylene glycol is equal to 32.045 mPa·s).^92,192^ Some hydrophobic DESs, based on fatty acids, also show low viscosities, which improves extraction efficiency.^15^ The extraction efficiency also depends on the p*K*_a_ of the HBD and the extracted compound. Metabolites with p*K*_a_ values that are lower than or similar to that of the HBD can form stronger hydrogen bond based interactions with the chloride anion in the HBA, compared to the HBD. This results in a higher extraction efficiency.^226,266^ Furthermore, if the extracted compound has more donor groups (carboxylic acid or hydroxyl groups), it can interact with HBAs even more strongly. The formation of hydrogen bonds is connected with solvation enthalpy, which is preferably exothermic. The isolation effectiveness is also driven by the extraction entropy change, which is an effect of breaking the DES structure via the solute. In the solvent structure, a cavity needs to be created to accommodate the solubilized compound. This fact is an explanation for the low extraction efficiencies of larger compounds—more energy is needed to create a cavity. It could be concluded that the isolation effectiveness depends mostly on the hydrogen bond creation between the compound and the chloride atom in the HBA, which is the most significant in extraction of polar chemicals.^266^

The same general rules and exceptions apply to flavonoids.^267^ Additionally, many flavonoids are soluble in alkaline solutions, so an extraction with acidic solvents is not preferable.^268^

### 3.3 Impact of the Extraction Method and Temperature

Natural compounds can be extracted using DESs through various methods, including assistance by ultrasound or microwaves or subcritical extraction techniques. These techniques are considered to be more effective than traditional stirring with heating because the plant cell walls can be more easily disrupted, which translates into a more efficient release of bioactive compounds.^269,270^ It also decreases the time needed for extraction.^74,153,190^ Unfortunately, microwave-assisted extraction can sometimes cause the degradation of sensitive metabolites.^194,271,272^

Considering the influence of the temperature of extraction, most of the processes are performed in the range 40–70 °C. A further increase in the temperature is risky, because some of the compounds, especially phenolics, tend to decompose at high temperatures, which affects their antioxidant properties.^88,194,273^ DESs can also react with extracted chemicals under such conditions, forming undesirable polymeric side products.^274^ On the other hand, the higher the temperature, the lower the viscosities of DESs and thus the better the mass transfer. Higher temperatures also enhance the cleavage of bonds between extracted compounds and the plant matrix.^275^

### 3.4 Impact of Water Content

Water content in the extraction media is another crucial element in the isolation of secondary metabolites from plants. Generally, DESs based on choline chloride have high viscosities, which have a negative effect on the extraction yield due to poor mass transfer. This problem can be solved by increasing the temperature of extraction (as was discussed in previous sections) or via the addition of water. In some cases, an addition of 25% (v/v) water to a DES can decrease the viscosity to the level of the water viscosity.^276^ Water can also increase the DES polarity, which can facilitate the extraction of some polar compounds, which creates a possibility for enhancing the selectivity of the extraction.^277−279^ Because of the high water concentration in the reaction system, the isolation of compounds with weak polarities was reduced, which was shown by Liu et al.: by increasing the water content to beyond 15%, the yield of curcuminoids extracted from *Curcuma longa* L. significantly decreased.^280^ On the other hand, too high a water percentage in the solvent can break the hydrogen bond structure of the DES, which leads to the deterioration of its properties and its abilities to extract bioactive compounds from the plant biomass.^273,276,281,282^ Apart from hydrogen bond breaking, there is also another possible mechanism that explains this phenomenon. An increase in the water content for the DES ChCl:ethylene glycol to above 30% (v/v) water creates aggregates in which the DES particles are suspended. The hydrogen bonds between the HBA and HBD compounds are preserved, but the eutectic properties of the solvent are significantly lower.^283^ A brief comparison of the extraction efficiencies, dependent on the water content, is presented in Table 9.^103,184,188,226^

**Table 9:** Yields of Extracted Compounds Using DESs, Dependent on Water Content

biomass	DES	extracted compd	water content [%]	yield [mg/g on mass of dry biomass]	ref
*Glycyrrhiza glabra* (roots)	ChCl:lactic acid	glycyrrhizic acid	0	28.00	(226)
	ChCl:lactic acid	glycyrrhizic acid	30	50.00	
*Fagopyrum esculentum* (sprouts)	ChCl:oxalic acid	orientin	0	0.44	(188)
	ChCl:oxalic acid	orientin	20	2.26	
*Larix decidua* (bark)	ChCl:urea	total flavonoid content	25	159.00	(184)
	ChCl:urea	total flavonoid content	50	305.00	
	ChCl:urea	total flavonoid content	75	275.00	
*Malus domestica* (fruit pomace)	ChCl:lactic acid	total phenolic content	10	3.90	(103)
	ChCl:lactic acid	total phenolic content	30	5.20	
	ChCl:lactic acid	total phenolic content	50	1.20	

## 4 Recovery of Isolated Chemicals from DESs

The major challenge in the extraction of bioactive chemicals is their recovery from DESs. Because of the negligible vapor pressures of these solvents, it is impossible to separate them from isolated compounds by distillation. There are a few developed methods, which were presented in recent literature, for example, macroporous resin adsorption, solid phase extraction, antisolvent method, and back extraction. The most applied method is adsorption on a macroporous resin, carried out in a packed column or by batch extraction. The recovery rate depends mainly on the type of the resin and its polarity, which should be similar to the polarity of the adsorbate. The most used resins are nonpolar (D-101, HP-20, X-5), middle polar (AB-8), and polar (HPD-600, XAD-7HP). Apart from the resin type, it is important to select the proper eluent, with an optimal desorption capacity.^284,285^ Solid phase extraction (SPE) is also one of the most popular methods of bioactive compounds recovery. It requires application of cartridges filled with sorbent, mainly reversed phase or hydrophilic–lipophilic balanced, which interacts noncovalently with the eluted analyte. It is one of the most effective methods, which allows recovery of analytes at a level above 90%.^188^ Even though solid phase extraction is considered an efficient method at the laboratory scale, recent findings in sorbent types (e.g., hierarchically porous silica monoliths providing a durable, high surface area that can be further functionalized with ligands) can open the way to industrial application of that technique.^286^ Another recovery method is adding antisolvent to the obtained DES extract, which breaks bonds between the HBA and the HBD. This causes the loss of DES properties and precipitation of the extracted chemicals as the antisolvent water is mostly applied, but also buffers.^287^ However, this method is not suitable for all isolated compounds.^288^ Back extraction is also a simple method for recovery of chemicals from DESs. It involves re-extraction of analytes with solvents which are immiscible with the DES, but the isolated compounds are soluble in them. The selection of the proper solvent increases the selectivity of re-extraction.^288^ Ethyl acetate, *n*-butanol, hexane, and acetone are the solvents most applied; however, they are not considered green chemicals, which makes this method not fully ecological. The economic issue, including the costs of additional solvent, its recovery, and recycling should also be considered. Table 10 presents a brief review on recovery of bioactive compounds from DESs.

**Table 10:** Recovery of Isolated Bioactive Compounds from DESs after Extraction

biomass	DES	extracted compds	recovery method	recovery rate [%]	ref
*Acanthopanax senticosus*	lactic acid:glycerol	flavonoids	resin absorption (D101)	55.76	(284)
	lactic acid:glycerol	flavonoids	resin absorption (AB-8)	77.10	
*Carthamus tinctorius* L.	ChCl:sucrose	carthamin	resin absorption (Diaion HP-20)	90.00	(285)
citrus fruit peels	ChCl:levulinic acid:*N*-methyl urea	polymethoxylated flavones	back extraction (ethyl acetate)	95.87	(288)
	ChCl:levulinic acid:*N*-methyl urea	hesperidin	back extraction (*n*-butanol)	86.32	
*Fagopyrum esculentum* Möench	ChCl:triethylene glycol	orientin	SPE (C18 cartridges)	97.81	(188)
	ChCl:triethylene glycol	rutin	SPE (C18 cartridges)	98.47	
*Ampelopsis grossedentata*	ChCl:glycerol	flavonoids	antisolvent (phosphate buffered saline)	86.75	(287)
*Ipomoea batatas* L.	ChCl:malic acid	flavonoids	resin absorption (AB-8)	85.46	(289)
Citri Reticulatae Pericarpium Viride	l-proline:urea	narirutin	resin absorption (SP825)	88.00	(290)
	l-proline:urea	hesperidin	resin absorption (SP825)	86.00	

## 5 Future Prospects

DESs are considered the solvents of the future that may be used in many fields. They can be applied not only as extraction media but also as drug carriers and adsorbents for harmful metals and organic molecules, H_2_S, and SO_2_, which are common byproducts in various processes. DESs may also be used as medium or catalysts in organic syntheses.^291^ There is an impressive number of publications and recently patented processes of extraction of natural products, with different types of DESs and systems containing DESs.^292^ The dramatic increase in the number of publications and patents in recent months (67 patent applications within 2022–2023, based on Espacenet) emphasizes the importance of the discussed topic. DESs have been demonstrated to have better extraction properties than many organic solvents, and they seem to be a promising alternative to them, due to the higher yields of extracted bioactive chemicals, preservation of their bioactivity, the mild extraction conditions, and their biodegradability. The proper selection of HBAs and HBDs in DESs allows for a natural, “tailor-made” solvent to be made for the best possible efficiency in the isolation of active compounds. According to the examples presented, the most frequently used solvents are based on organic acids of natural origin, especially lactic acid, which is one of the most suitable HBDs for obtaining high yields for many chemicals. In order to maximize the yields of isolated chemicals, extraction with the assistance of ultrasound or microwaves is recommended. A combination of these techniques with green solvents makes the extraction process more sustainable and consistent with green chemistry rules. Undeniably, studies regarding extractions with DESs are constantly being developed.

This review presented the recent results achieved with DESs, especially solvents based on choline chloride as the HBA. However, there is a growing trend to modify DESs and their properties. Many researchers focus on hydrophobic DESs, which have a great potential for the extraction of less polar compounds, e.g., carotenoids, sulforaphane, and ginkgolic acids.^240,293,294^ The other tendency is to prepare ternary DESs and NADESs, composed usually of choline chloride, diol or polyalcohol, and acid, e.g., choline chloride/lactic acid/ethylene glycol or choline chloride/butanediol/citric acid, where the acidity can be tuned by using different amounts of acids.^95,98^ In addition, the development of switchable systems is receiving more attention. With the use of such a system, an efficient and selective separation of compounds with different polarities is possible. For instance, rosmarinic acid and carnosic acid were extracted from rosemary, using a thermo-switchable system composed of a DES and an IL, namely ChCl:levulinic acid and [BMIM]PF_6_. Due to the different polarities of the solvents, the selective extraction of compounds was possible: the IL phase was rich with less polar carnosic acid and, in the DES phase, polar rosmarinic acid was present.^295^ Thermo- and pH-switchable DESs are also promising solvents in green extraction. For example, by increasing the pH value, it is possible to create a homogeneous hydrophobic DES–water mixture, and addition of acid after the extraction separates the DES and aqueous phases.^296,297^ This is a promising approach to the problem of DES separation after extraction. This problem can also be solved by applying magnetic DESs containing metal oxides or salts, e.g., Fe_3_O_4_, TiO_2_, FeCl_3_, NiCl_2_, and ZnCl_2_. These solvents can be separated from the aqueous solution by using a magnetic field. Furthermore, there is evidence from the literature that magnetic DESs are more effective extraction media than traditional DESs.^298,299^ From the separation of the solute after extraction point of view, deep eutectic solvent molecularly imprinted polymers (DES-MMIPs) have great potential. For example, MMIPs with choline choride/caffeic acid/formic acid used as functional monomers were obtained and successfully used in the extraction and determination of hormones from lotion.^300^ When new deep eutectic systems are designed for extraction, their price must be taken into account. Classic DESs are valued for their properties but also for the simple way of obtaining them and their relatively low price. Modern systems require more work and are more expensive. In the final calculation, however, the entire extraction process, the entire economy, the amount of waste, and, above all, the yield of the isolated, pure product must be taken into account.

The most important difficulties in the application of DESs in industrial extraction processes are the high viscosities of many of them, as well as the separation of the solute after extraction. Design of DESs with lower viscosities by selecting new, biodegradable, natural, and cheap HBAs and HBDs, and mixing them in appropriate ratios, seems to be one of the challenges on the way to the industrial use of these solvents. The viscosity problem can also be solved by the addition of water to the extraction system or an increase of temperature. The amount of added water should be thoroughly examined, in order to maintain the properties of DESs. According to the literature, usually addition of up to 30% of water is safe, but there are also publications where even 50% of water was used, especially in ternary systems.^95^ Another issue is an efficient separation of the extracted compounds from the solvents, which is crucial in the application of extracts in pharmaceuticals, cosmetics, or foods. As previously mentioned, application of switchable systems, ternary systems, or magnetic DESs can be a solution for that problem. However, a simple, more economical and efficient method of solute recovery and solvent recycling should be elaborated. Finally, even though most DESs are considered biocompatible, nontoxic, and environmentally friendly, their application in the extraction of cosmetic, food, and pharmaceutical ingredients needs an individual, thorough safety examination.
